# Development of a loop-mediated isothermal amplification (LAMP) method for specific detection of *Mycobacterium bovis*

**DOI:** 10.1371/journal.pntd.0008996

**Published:** 2021-01-25

**Authors:** Thoko Flav Kapalamula, Jeewan Thapa, Mwangala Lonah Akapelwa, Kyoko Hayashida, Stephen V. Gordon, Bernard Mudenda Hang' ombe, Musso Munyeme, Eddie Samuneti Solo, Precious Bwalya, Mirriam Ethel Nyenje, Aki Tamaru, Yasuhiko Suzuki, Chie Nakajima

**Affiliations:** 1 Division of Bioresources, Research Center for Zoonosis Control, Hokkaido University, Sapporo, Hokkaido, Japan; 2 Division of Collaborations and Education, Research Center for Zoonosis Control, Hokkaido University, Sapporo, Hokkaido, Japan; 3 International Collaboration Unit, Research Center for Zoonosis Contsrol, Hokkaido University, Sapporo, Hokkaido, Japan; 4 School of Veterinary Medicine, University College Dublin, Dublin, Ireland; 5 School of Veterinary Medicine, University of Zambia, Lusaka, Zambia; 6 Africa Center of Excellence for Infectious Diseases of Humans and Animals, University of Zambia, Lusaka, Zambia; 7 Department of Pathology and Microbiology, University Teaching Hospital, Ministry of Health, Lusaka, Zambia; 8 Community Health Sciences Unit, National Tuberculosis Reference Laboratory, Lilongwe, Malawi; 9 Department of Microbiology, Osaka Institute of Public Health, Osaka, Japan; Oregon State University College of Veterinary Medicine, UNITED STATES

## Abstract

Bovine tuberculosis (TB) caused by *Mycobacterium bovis* is a significant health threat to cattle and a zoonotic threat for humans in many developing countries. Rapid and accurate detection of *M*. *bovis* is fundamental for controlling the disease in animals and humans, and for the proper treatment of patients as one of the first-line anti-TB drug, pyrazinamide, is ineffective against *M*. *bovis*. Currently, there are no rapid, simplified and low-cost diagnostic methods that can be easily integrated for use in many developing countries. Here, we report the development of a loop-mediated isothermal amplification (LAMP) assay for specific identification of *M*. *bovis* by targeting the region of difference 4 (RD4), a 12.7 kb genomic region that is deleted solely in *M*. *bovis*. The assay's specificity was evaluated using 139 isolates comprising 65 *M*. *bovis* isolates, 40 *M*. *tuberculosis* isolates, seven *M*. *tuberculosis* complex reference strains, 22 non-tuberculous mycobacteria and five other bacteria. The established LAMP detected only *M*. *bovis* isolates as positive and no false positives were observed using the other mycobacteria and non-mycobacteria tested. Our LAMP assay detected as low as 10 copies of *M*. *bovis* genomic DNA within 40 minutes. The procedure of LAMP is simple with an incubation at a constant temperature. Results are observed with the naked eye by a color change, and there is no need for expensive equipment. The established LAMP can be used for the detection of *M*. *bovis* infections in cattle and humans in resource-limited areas.

## Author summary

Although bovine tuberculosis in humans has been eliminated in developed countries, the disease remains a challenge in many developing countries. Routine laboratory methods used to identify tuberculosis (TB) in high-burden countries do not distinguish between the two main causes of TB in humans, namely *Mycobacterium tuberculosis* and *M*. *bovis*. In addition, *M*. *bovis* is naturally resistant to one of the first-line drugs used to treat TB called pyrazinamide; therefore, accurate diagnosis of *M*. *bovis* is important for proper selection of anti TB drugs. In cattle, surveillance for *M*. *bovis* infection is important to obtain data on bovine TB burden and hence provide a basis for the establishment and/or improvement of control programs. In this study, a loop-mediated isothermal amplification (LAMP) based method was developed to identify *M*. *bovis*. This LAMP method detected *M*. *bovis* within 40 minutes following incubation at constant temperature (66°C) in a battery-powered incubator and results could be read with the naked eye following development of a color change. Our results elaborate a rapid and low-cost LAMP based method for detection and surveillance of *M*. *bovis* infection in cattle and humans in resource-limited, endemic areas.

## Introduction

*Mycobacterium tuberculosis*, the archetypal member of the *M*. *tuberculosis* complex (MTC), is the principal causative agent of tuberculosis (TB) in humans [1]. Additionally, *Mycobacterium bovis*, which is the main causative pathogen of bovine TB, also causes TB disease in humans which is termed 'zoonotic TB'. In both hosts, *M*. *bovis* infections pose serious challenges. For instance, in cattle annual economic losses are estimated at US$3 billion [2]. The losses incurred include condemnation of carcasses at slaughterhouses, mortalities and costs involved in the implementation of disease control measures [2,3]. These challenges are immense mostly in developing and high burdened countries in comparison to developed countries. In the latter, appropriate food hygiene measures such as milk pasteurization and "test and slaughter" control programs to remove infected animals have helped reduce the disease burden. However, this is not the case in developing countries due to a lack of compliance and absence of associated control measures due to financial constraints [4]. In humans, the zoonotic transmission of *M*. *bovis* infection from animals poses challenges to standard TB diagnosis and treatment [5]. *M*. *bovis* is innately resistant to pyrazinamide (PZA), a critical first line anti-TB drug that plays a unique role in shortening the duration of TB treatment [6]. Hence, if PZA is included in a patient's regimen with *M*. *bovis* infection, the drug is ineffective while the patient is exposed to the potential side effects such as hepatotoxicity and polyarthralgia [7]. However, if *M*. *bovis* is detected during routine TB diagnosis, then the standard TB regimen can be optimized [5].

In developing countries, routine TB diagnosis is still achieved through clinical symptoms, chest radiography and sputum smear microscopy. Additionally, in referral hospitals or laboratories, BACTEC MGIT culture (Becton Dickinson (BD), USA) or GeneXpert (Cepheid, Sunnyvale, CA, USA) systems are also used. Unfortunately, these methods do not have the capacity to distinguish MTC to the species level [8]. Mycobacterial culture, being the gold standard method for TB diagnosis, takes several weeks to months to read results and thus is unable to provide timely diagnosis [9]. Testing technologies capable of identifying *M*. *bovis* have varying limitations prohibiting easy integration in resource-limited areas. For instance, biochemical based tests such as nitrate reduction and niacin accumulation [10] rely on mycobacterial growth and are hence time consuming. Nucleic acid amplification tests (NAAT) such as PCR targeting single nucleotide polymorphisms in the *pncA* and *oxyR* genes [11] involve complex procedures, use of expensive equipment and standardized laboratory infrastructure that constrains their use in many developing countries [12].

Lack of rapid, simple and low-cost methods for specific differentiation of MTC species has, to some extent, been caused by the high genetic homogeneity shared among MTC species which show 99% identity at the nucleotide level and with identical 16S rRNA sequences, a molecular marker commonly used in microbiology to distinguish species [13]. Indeed, this high level of genetic identity has led to the MTC species being recently proposed as the same species [14]. Nevertheless, MTC comparative genomic studies have revealed the distinct evolutionary trajectory of the MTC species, traced by the deletion of specific regions [15]. For instance, region of difference 4 (RD4) is a 12.7kb locus that encompasses 11 genes, from Rv1506c - Rv1516c in *M*. *tuberculosis*, which is deleted from *M*. *bovis* [15]. Therefore, the RD4 deletion could be utilized as a molecular marker to differentiate *M*. *bovis* from *M*. *tuberculosis*. Indeed, PCR approaches have previously been reported that target RD4 for differentiation of *M*. *bovis* from *M*. *tuberculosis* [16,17]. Despite being specific for detecting *M*. *bovis*, these PCR-based methods are not easily adopted for use in resource limited areas because of the aforementioned constraints mentioned above. Thus, a rapid, simple and low cost diagnostic tool is required in such areas for specific detection of *M*. *bovis* that can be easily incorporated into existing systems [18].

Loop-mediated isothermal amplification (LAMP) is a single tube NAAT technique that was developed by Notomi *et*. *al*. in 2000 [19]. LAMP doesn't require equipment such as a thermo-cycler or electrophoresis system since DNA amplification occurs under a constant temperature, and the results are visible to the naked eye. LAMP is recommended for use in resource limited areas due to its simplicity, rapidity, sensitivity and use of low cost equipment [20]. LAMP has been reported extensively for detecting several infectious diseases such as malaria [21], TB [22,23] and African trypanosomiasis [24]. In this study, we have developed and utilized a LAMP based technology for specific detection of *M*. *bovis* by targeting the deletion of RD4.

## Materials and methods

### Ethics statement

The ethical approval to conduct this study was granted by The University of Zambia Biomedical Research Ethics Committee for samples from Zambia and National Health Science Research Committee (NHSRC), Ministry of Health and Population, Lilongwe, Malawi for samples from Malawi. The Zambia National Health Research Ethics Committee and the Department of Animal Health and Livestock Development in Malawi approved the transfer of mycobacterial DNA to Hokkaido University (Japan) for molecular analysis.

### Samples and DNA extraction

Extracted DNA from 139 mycobacteria and non-mycobacteria were used to determine the specificity of our LAMP assay comprising of seven reference MTC strains, twenty-two reference non-tuberculous mycobacteria (NTM) strains, five reference non-mycobacteria and 105 clinical or field MTC isolates or TB lesion specimens as shown in Tables 1 and S1. We included *M*. *caprae* and *M*. *orygis*, two other important causative agents of zoonotic TB associated with cattle, as they are pyrazinamide susceptible and thus differentiation from *M*. *bovis* was crucial. NTMs were selected as representative mycobacteria that would be useful for differential diagnosis relative to *M*. *bovis* infections. A further five bacterial species, that are agents of respiratory infections, were also included to represent general pathogens that would be useful for differential diagnosis of TB in humans.

**Table 1:** Bacteria used in this study to determine specificity.

	Bacterial species	Sample ID
MTC reference strains	*M*. *bovis* BCG	172-Tokyo
	*M*. *tuberculosis*	H37Rv
	*M*. *tuberculosis*	H37Ra
	*M*. *africanum*	KK 13–02
	*M*. *caprae*	EPDC01 ^a^
	*M*. *orygis*	NepR1 ^b^
	*M*. *microti*	ATCC 19422
MTC clinical isolates	*M*. *bovis*	65 isolates ^c^
	*M*. *tuberculosis*	40 isolates ^c^
NTM reference strains	*M*. *abscessus*	JATA 63–01
	*M*. *asiaticum*	KK 24–01
	*M*. *avium*	JATA 51–01
	*M*. *chelonae*	JATA 62–01
	*M*. *fortuitum*	JATA 61–01
	*M*. *gastri*	KK 44–01
	*M*. *gordonae*	JATA 33–01
	*M*. *intermedium*	JATA 9H-01
	*M*. *intracellulare*	JATA 52–01
	*M*. *kansaii*	KK 21–01
	*M*. *lentiflavum*	JATA 9N-01
	*M*. *malmoense*	JATA 47–01
	*M*. *marinum*	JATA 22–01
	*M*. *mucogenicum*	JATA 9P-01
	*M*. *nonchromogenicum*	JATA 45–01
	*M*. *peregrinum*	JATA 61–01
	*M*. *scrofulaceum*	JATA 31–01
	*M*. *shimodei*	JATA 54–01
	*M*. *simiae*	KK 23–01
	*M*. *smegmatis*	JATA 64–01
	*M*. *szulgai*	JATA 32–01
	*M*. *terrae*	JATA 46–01
Other bacteria	*Streptococcus pneumoniae*	NBRC 102642
	*Klebsiella pneumoniae*	NBRC 3318
	*Pseudomonas aeruginosa*	NBRC 12689
	*Staphylococcus aureus*	NBRC 100910
	*Mycoplasma pneumoniae*	NBRC 14401

a reference [31]

b reference [32]

c More information in S1 Table.

Sixty-five isolates of *M*. *bovis* and forty of *M*. *tuberculosis* were used. Fifty-one isolates grown on Ogawa medium (Kyokuto Pharmaceutical Industrial Co., Ltd., Tokyo, Japan) and identified as *M*. *bovis* by a MTC-discrimination multiplex PCR [25] and spoligotyping [26] were collected from cattle and wild lechwe antelope from 2004 to 2009 in Zambia [27]. Twenty clinical samples grown in mycobacterium growth indicator tubes (MGIT) (Becton, Dickinson and Company, NJ, USA) were obtained at the University teaching hospital in Lusaka, Zambia during 2011 to 2016 [28]. Four of those were confirmed as *M*. *bovis* and 16 were *M*. *tuberculosis* by Capilia TB (Tauns Laboratories Inc., Shizuoka, Japan) and spoligotyping. Ten samples were collected from cattle suspected of TB during routine postmortem at Lilongwe cold storage abattoir in Lilongwe Malawi in November 2019. Briefly, samples were homogenized, decontaminated in 4% NaOH for 15 minutes, followed by neutralization in sterile phosphate buffer saline (PBS) and centrifuged at 3200 g for 20 min at 6°C. Part of the pellets was re-suspended in PBS and DNA was isolated directly by heating at 95°C for 15 minutes and immediately cooling at -20°C for 30 minutes. Confirmation as *M*. *bovis* was done at Hokkaido University, Japan using Mycobacterium specific PCR IS*6110* [29], MTC-discrimination multiplex PCR and spoligotyping. The other 24 *M*. *tuberculosis* isolates were collected in Osaka, Japan during 2000 to 2009 [30] and grown on Ogawa medium. Bacterial DNA from colonies on solid medium was extracted as previously described [29]. For liquid medium cultures, 500 μl of the MGIT contents were taken to a cryotube and DNA was extracted by boiling at 95°C for 15 minutes. All extracted DNA were stored at -30°C.

### Primer design and screening

The location of RD4 deletion was determined by sequence alignment of *M*. *bovis* AF2122/97 genome sequence (GenBank accession No.: LT708304.1) against *M*. *tuberculosis* H37Rv genome sequence (GenBank accession No.: AL123456.3) using Molecular Evolutionary Genetics Analysis (MEGA) version 7 software (https://www.megasoftware.net/ Pennsylvania State University, USA). The target sequence was chosen from upstream through to downstream flanking the RD4 deletion. LAMP primers were designed using the online Primer Explorer V5 software (http://primerexplorer.jp/lampv5e/index.html, Eiken Chemical, Tokyo, Japan). A set of primers comprised of 4 primers, namely: outer primers (Forward outer primer-F3 and Backward outer primer-B3) and inner primers (Forward inner primer-FIP and Backward inner primer-BIP). Loop primers (Forward loop primer-FLP and Backward loop primer-BLP) were designed and added for the best performing primer set that was selected and optimized. In order to determine a set of primers specific for detection of *M*. *bovis*, primer design properties and position of RD4 deletions were manually adjusted with reference to the "Advanced primer design" manual [33]. Designed LAMP primers were screened by observing the specificity, cross-reactivity and reaction speed in order to select the best performing primer set. All primers were synthesized by Life Technologies Japan Ltd. (Tokyo, Japan).

### Optimization of LAMP reaction

LAMP reactions were performed according to the procedures in Pandey et al [22]. Reaction tubes were incubated at 64°C for 120 minutes in a Loopamp real-time turbidimeter (LA-200: Teramecs Co. Ltd., Kyoto, Japan). *M*. *bovis* BCG Tokyo 172 genomic DNA was used as positive control while *M*. *tuberculosis* H37Rv DNA and double distilled water (DDW) were both negative controls for every run. Positive results were indicated by rising curve(s) of turbidity greater than 0.1 threshold in the real time turbidimeter (LA-200) and visual inspection of the color change by colori-fluorometric indicator (CFI) [24]. In order to improve LAMP performance, modifications to three parameters of the LAMP reaction were screened. The incubation temperature was screened at 1°C intervals from 60°C to 67°C; the concentration of loop primers was evaluated at 5.0 μM, 4.2 μM, 3.6 μM and 2.8 μM. Inner primers (FIP/BIP) and outer primers (F3/B3) were kept fixed at 4.8 μM and 0.6 μM, respectively. The primer mixture's final volume per reaction was adjusted in 0.25 μl intervals from 2.0 μl to 2.75 μl.

### Specificity and sensitivity analysis

Using the optimized conditions, LAMP specificity was evaluated using bacterial genomic DNAs listed in Tables 1 and S1. To assess the specificity, the results of the newly established LAMP were compared to the initial identification and confirmation results using other conventional methods (S1 Table). Sensitivity of our new LAMP system was evaluated using serially diluted *M*. *bovis* BCG Tokyo 172 genomic DNA, 2.5pg/μl, 250fg/μl, 25fg/μl, 20fg/μl and 15fg/μl (5pg ~ 30fg/reaction). DNA concentration was measured using a Qubit 3 Fluorometer (Thermo Fisher Scientific, MA, USA) according to the manufacturer's instructions. Reactions were performed in duplicates and repeated 4 times (8 reactions). All reactions were monitored up to 120 min after the beginning of incubation by the LA-200 (Teramecs Co. Ltd.). In the specificity study, the cut-off point to determine positivity and negativity was set to 40 minutes.

### Multiplex PCR reaction

Multiplex PCR targeting RD4 [16] was performed to compare its sensitivity to our new LAMP assay. The protocol of multiplex PCR was slightly modified, as follows: 4 μl 5× Go Taq buffer (Promega Co., WI, USA); 0.6 μl of 25mM MgCl_2_; 0.5 μl of 25mM dNTP mix; 0.5 μl of 10 μM each primer; common forward primer—CBS1 (5'-TTCCGAATCCCTTGTGA-3'); *M*. *bovis* specific reverse primer—CBS2 (5'-GGAGAGCGCCGTTGTA-3') and *M*. *tuberculosis* specific reverse primer—CBS3 (5'-AGTCGCCGTGGCTTCTCTTTTA-3'); 0.2 μl of 5U/μl GoTaq DNA polymerase (Promega Co.); 1 μl of template DNA; and finally double-distilled water (DDW) to make up to a final volume of 20 μl. The cycling parameters consisted of an initial denaturation at 94°C for 5 min, followed by 30 cycles of denaturation at 94°C (1 min), annealing at 52°C (1.5 min), and extension at 72°C (1 min), with a final elongation step at 72°C for 5 min. The amplification products were analyzed by gel electrophoresis at 100v for 20 min. The predicted PCR products were 168 bp (*M*. *bovis)* and 337 bp (*M*. *tuberculosis*).

### Confirmation of LAMP products

In-silico LAMP fragment analysis (http://creisle.github.io/creisle.lamprflp/) was performed to predict LAMP product sizes. Then, LAMP products were digested by restriction enzyme *Eco*RI (New England Biolabs, MA, USA). Enzymatic digestion reaction mixture comprised of 1 μl of LAMP product, 1 μl of *Eco*RI, 2 μl of 10x enzyme buffer (New England Biolabs) and 16 μl of DDW. The mixture was incubated at 37°C for 60 min and the resultant products analyzed by 2% agarose gel electrophoresis stained with Gel-Red (Biotium, Inc., CA, USA).

## Results

### LAMP primers screening and selection

**Fig 1:**
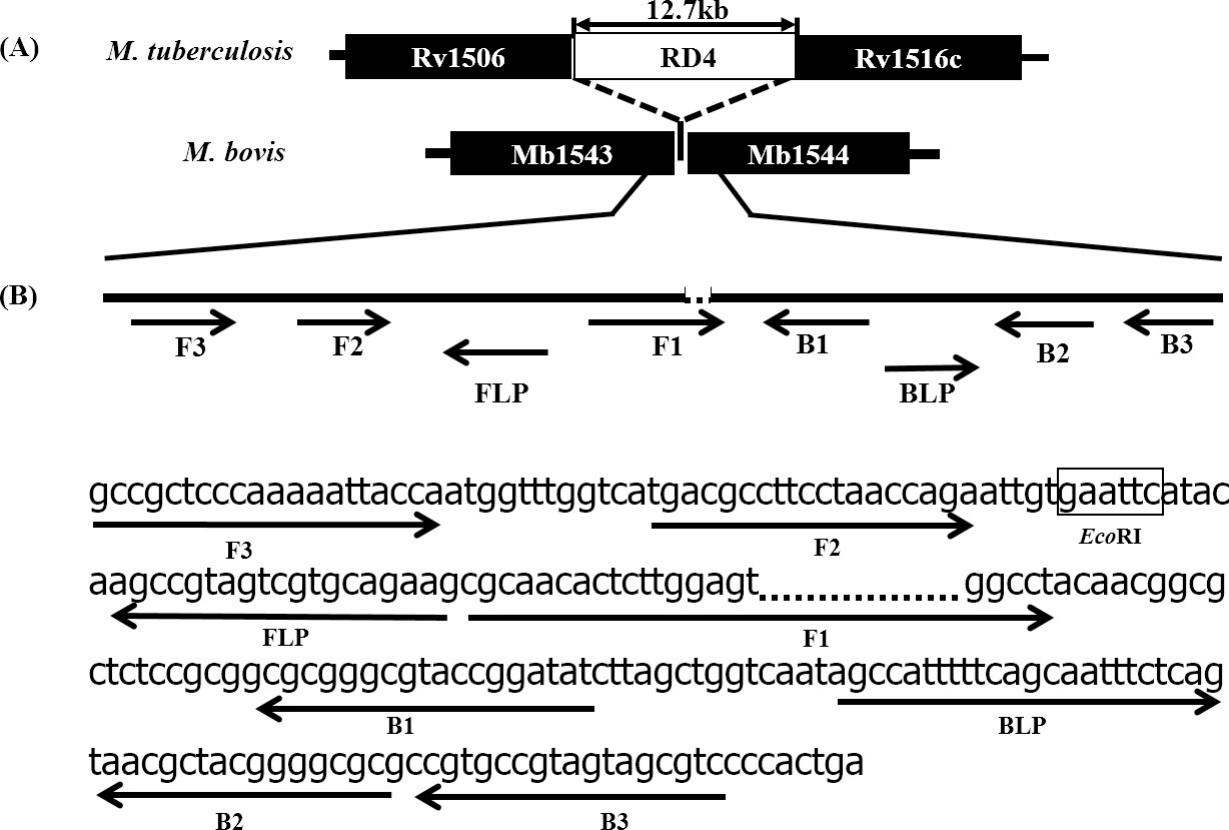
LAMP primers for specific detection of *M*. *bovis* targeting the RD4 flanking region. (A) Position of the RD4 deletion on *M*. *bovis* genome, shown with reference to *M*. *tuberculosis*. The RD4 locus is present in *M*. *tuberculosis* but deleted from *M*. *bovis*. (B) The target sequence, individual primers, annealing positions and their directions of the set of primers with RD4 deletion at 3-prime region of F1 primer are shown. Restriction enzyme *Eco*RI target sequence is indicated in a box between F2 and FLP.

More than 40 sets of primers targeting the RD4 deletion were designed and screened. Primer design properties and position of the RD4 deletion were manually configured while screening for the best performing set of primers for specific detection of *M*. *bovis*. A primer set where the F1 primer 3-prime section was located across the RD4 deletion-junction showed high specificity and repeatability and was selected for further optimization (Table 2). The target sequence and primer binding positions are shown in Fig 1.

**Table 2:** Nucleotide sequences and sizes of established LAMP primers.

Primer	Length	Sequence (5'-3')
F3	20	GCCGCTCCCAAAAATTACCA
B3	18	GACGCTACTACGGCACGG
FIP	41	AGGCCACTCCAAGAGTGTTGCG-TGACGCCTTCCTAACCAGA
BIP	35	GCGCGGGCGTACCGGATAT-GCGCCCCGTAGCGTTA
FLP	19	CTTCTGCACGACTACGGCT
BLP	24	AGCCATTTTTCAGCAATTTCTCAG

FIP primer consisted of F1c and F2; BIP primer consisted of B1 and B2c.

### Optimization of LAMP assay

Different incubation temperatures at 1°C intervals from 60°C to 67°C were evaluated. An increase in temperature gave an improved detection speed and specificity. However, at 67°C the LAMP assay sensitivity decreased; thus, the optimal temperature was determined as 66°C. The effect of loop primers was tested at varying concentrations (Table 3). Upon adding 5.0 μM of loop primers to the primer mixture, the assay's detection speed improved with *M*. *bovis;* however, false positives from *M*. *tuberculosis* were observed. Reducing the concentration of loop primers (4.2 μM) improved the specificity. We observed that by using the lowest concentration of loop primers (2.8 μM) LAMP specificity improved, but sensitivity was reduced. Therefore, the optimal concentration of loop primers was determined as 3.6 μM. Primer mixture volume was optimized in the same way and a 2.25μl/reaction volume was determined to be optimal. With this combination of conditions, no false positives with *M*. *tuberculosis* were observed up to 120 min incubation. Considering these adjustments, the final LAMP reaction conditions for our new assay were as follows: reaction temperature of 66°C; 2.25 μl of final primer mixture comprising of 0.6 μM of each outer primer, 4.8 μM of each inner primer, 3.6 μM of each loop primer; 20 mM Tris-HCl (pH 8.8, BIORAD); 10 mM KCl; 10 mM (NH_4_)_2_SO_4_; 0.1% Tween20; 6 mM MgSO_4_; 0.8 M betaine; 1.25 mM dNTP; 8U *Bst* DNA polymerase (Nippon Gene Co., Japan); 1 μl of CFI [24]; 2 μl of extracted DNA as a template and DDW up to a final volume of 25 μl.

**Table 3:** Optimization of primer concentrations and ratios in LAMP reaction.

Loop Primer concentration								
	5.0μM		4.2μM		3.6μM		2.8μM	
DNA concentration	BCG	H37Rv	BCG	H37Rv	BCG	H37Rv	BCG	H37Rv
5pg	21.0±1.3	82.1±5.9	23.8±0.6	95.3±2.6^a^	24.8±0.6	NA	35.2±3	NA
500fg	24±1.7	NT	24.9±1.2	NT	31.6±2.1	NT	39.9±10.1	NT
50fg	27.1±0.9	NT	30.0±3.1	NT	36.1±4.0^b^	NT	NA	NT
NC	NA		NA		NA		NA	
Primer volume quantity								
	2.0μl		2.25μl		2.5μl			
DNA concentration	BCG	H37Rv	BCG	H37Rv	BCG	H37Rv		
5pg	37.6±1.6	117.3^c^	32.8±4	NA	23.8±2.1	101.3±10.1^d^		
500fg	41.9±6.2	NT	35.3±2.1	NT	31.9±7.0			
50fg	70±15.7^e^	NT	45.1±9.0^f^	NT	34.4±1.9			
NC	NA		NA		NA			

LAMP reactions were performed six times (duplicate x 3). Results are shown in time (min.) to be positive presented as mean ± standard deviation. BCG: *M*. *bovis* BCG 172 Tokyo; H37Rv: *M*. *tuberculosis* H37Rv; NA: No amplification observed within 120 min of reaction; NT: Not tested; NC: Negative control (DDW); NA: No Amplification

a 2 of 6 reactions were positive

b 5 of 6 reactions were positive

c 1 of 6 reactions were positive

d 4 of 6 reactions were positive

e 3 of 6 reactions were positive

f 4 of 6 reactions were positive

### Specificity of LAMP assay

An analysis of LAMP specificity was performed by testing 139 DNA samples from bacteria listed in Tables 1 and S1. The assay detected all 65 *M*. *bovis* isolates as positive, while all other bacteria tested negative. There were no false positives, which is particularly relevant when considering we tested against *M*. *tuberculosis* isolates and other MTC species. All isolates detected as *M*. *bovis* by our newly developed LAMP system were in agreement with their initial identification of the samples as *M*. *bovis* by other conventional methods (S1 Table).

### Sensitivity of LAMP assay and multiplex PCR

**Fig 2:**
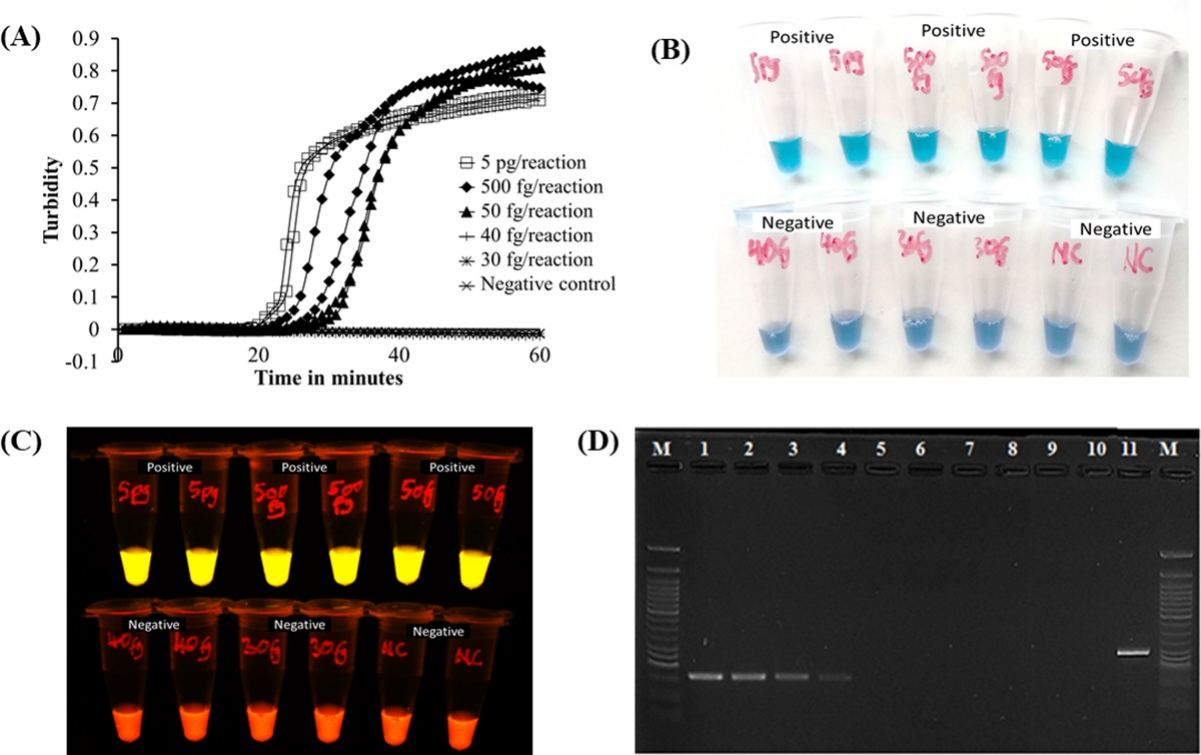
Sensitivity of established LAMP assay and multiplex PCR. A) LAMP results observed by rising curves of turbidimeter. B) LAMP results observed by the naked eye under natural light; the colour shifts from violet to sky blue for positive samples. C) Under LED light, the color change is from orange to light yellow for positive samples. D) The gel electrophoresis result of the multiplex PCR [16]. Lane M, 50bp DNA marker (New England Biolabs); lanes 1–9, *M*. *bovis* BCG Tokyo 172 genomic DNA 500pg, 50pg, 20pg, 5pg, 500fg, 50fg, 40fg, 30fg, 20fg/reaction; lane 10, Negative Control (DDW); lane 11, *M*. *tuberculosis* H37Rv.

Sensitivity analysis of our newly established LAMP was performed by determining limit of detection using diluted *M*. *bovis* BCG Tokyo 172 genomic DNA. The detection limit of our new LAMP was 50fg/reaction, equivalent to 10 copies of *M*. *bovis* genome, in 40 minutes. These results were observed both on the LAMP turbidimeter (Fig 2A) as well as visually by the naked eye under natural light (Fig 2B) and under LED light (Fig 2C). Comparatively, multiplex PCR [16] performed on the same samples detected up to 5pg/reaction (Fig 2D).

### Confirmation of LAMP products

**Fig 3:**
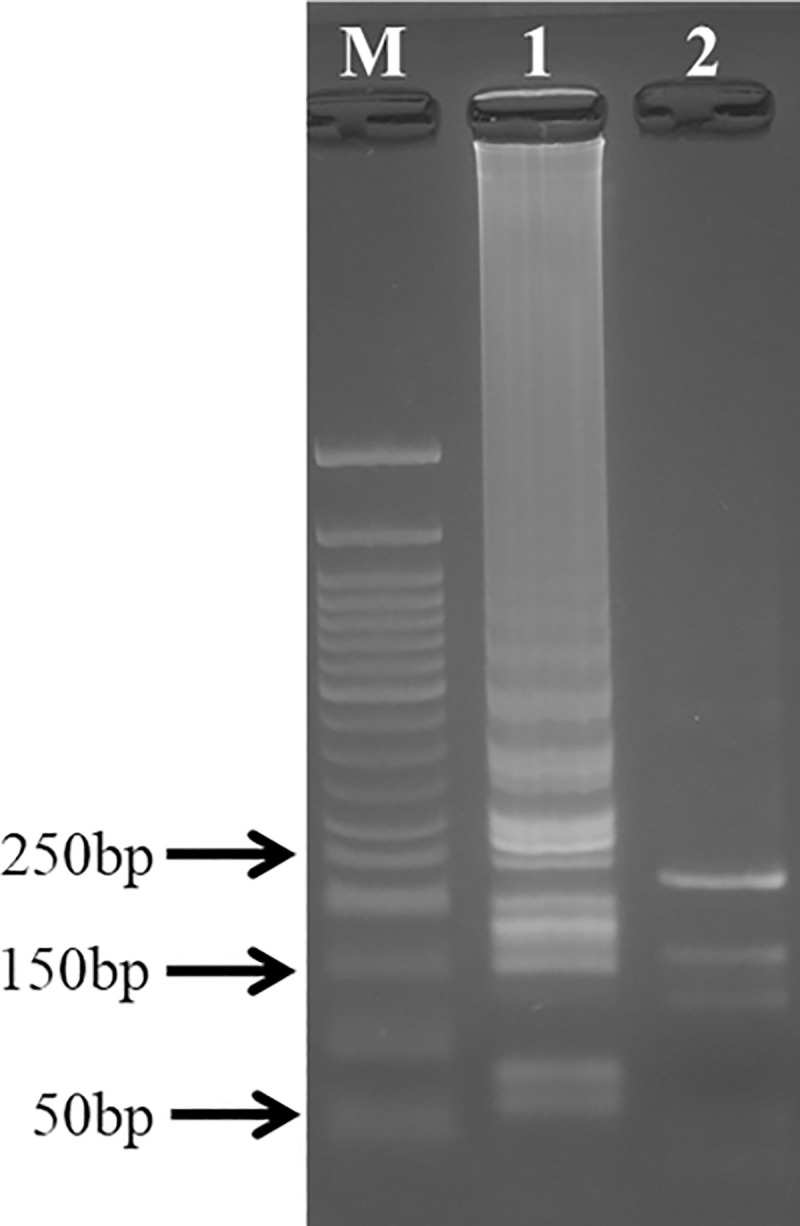
Enzymatic digestion of target sequence by restriction enzyme *Eco*RI. Lane M, 50bp DNA maker (New England Biolabs). Lane 1, LAMP products without the restriction enzyme. Lane 2, LAMP products digested by *Eco*RI. The predicted product sizes were 124bp, 169bp and 239bp.

LAMP products were digested by restriction enzyme *Eco*RI to confirm successful amplification of the target sequence. Digested LAMP products gave three fragments of 124 bp, 169 bp and 239 bp (Fig 3) as predicted in-silico (S1 Fig).

## Discussion

Bovine tuberculosis represents a potential zoonotic threat to humans in developing countries, but its control has long been neglected. There is substantial evidence suggesting that developing countries bear the highest burden of zoonotic TB due to existence of multiple risk factors maintaining the circulation of the disease in livestock [34]. In Africa a range of 0%-37.7% of all TB cases in humans are estimated to be caused by *M*. *bovis* [34]. However, detection of *M*. *bovis* is seldom performed, largely because routine TB diagnostic methods do not have the capacity to differentiate *M*. *bovis* from other MTC. Methods capable of delineating MTC species, such as PCR or culture-based methods, are time consuming, complicated, and prohibitively expensive and hence not easily adopted for widespread use. Consequently, all TB suspected cases adopt the standard *M*. *tuberculosis* treatment regimen that includes pyrazinamide, even though this approach has significant shortcomings in cases of *M*. *bovis* infection [35].

In this study, we describe the development of a LAMP-based method for specific identification of *M*. *bovis* by targeting the RD4 deletion. Thus, our newly established LAMP assay is important not only for guiding appropriate choice of antimicrobials against zoonotic TB but also for surveillance purposes and disease control. The developed LAMP method accurately identified all *M*. *bovis* isolates, while no false positive amplifications were observed with other MTC species (Tables 1 and S1). One cattle sample on Ogawa medium was heavily contaminated with other bacteria, and *Pseudomonas* sp. sequence was detected by 16S rRNA gene sequencing (C-43, S1 Table); however, the LAMP reaction became positive in less than 40 min in both duplicated test tubes. These results show that our established LAMP is highly specific for detecting *M*. *bovis* even in contaminated samples. Furthermore, the results show evidence for 'proof of concept' in using the RD4 deletion as a molecular signature for specific identification of *M*. *bovis* amongst other MTC species. Elsewhere, LAMP based methods have been reported for differentiating *M*. *bovis* from MTC targeting *mbp70* [36] and *mtp40* genes [37]. However, the specificity to detect *M*. *bovis* only is open to challenge because *mbp70* is also present in other MTC species [38], while *mtp40* is not present in all *M*. *tuberculosis* strains [39]. In view of this, our LAMP is reliable for the identification and differentiation of *M*. *bovis* from MTC because it targets RD4 which is the best target for this purpose as all *M*. *bovis* strains lack RD4 [15,40].

We used a variety of *M*. *bovis* and *M*. *tuberculosis* samples in our validation, encompassing *M*. *bovis* derived from different animal species, clinical samples from different countries, and *M*. *tuberculosis* strains from different lineages and a variety of spoligotypes (S1 Table). All samples gave consistent LAMP results. Moreover, our method can be performed using DNA extracted directly from cattle specimens and a variety of growth media, liquid (MGIT) and solid culture (Ogawa medium). Regardless of the sample source, the performance of our LAMP assay was the same in all cases. The ability to use simply heat-killed MGIT culture is very advantageous in this regard since the medium is now widely used in developing countries, with MGIT positive culture growth indicated within 7 to 14 days. Hence this LAMP could be incorporated with standard MGIT to confirm and speciate positive growth results, allowing diagnosis at the earliest possible time.

We also performed the established LAMP system using larger amounts of mycobacterial DNA (50pg/reaction, an equivalent to smear positive sputum+++) [41] in order to evaluate the applicability on samples with an unknown number of bacterial cells/DNA concentration. We observed no false amplification of *M*. *tuberculosis* until a reaction time of 92 minutes. Thus, the results show the ability of our established LAMP to specifically identify *M*. *bovis*. Despite the false amplification of *M*. *tuberculosis* when larger amounts of DNA were used, our LAMP reaction time could be shortened to 40 minutes to ensure specific identification of *M*. *bovis*. The established LAMP is highly sensitive, with the assay detecting as few as to 10 copies of *M*. *bovis* genomic DNA. In comparison to previous reports, a LAMP system targeting RD1 deletions for identification of *M*. *bovis* BCG required more than 200 copies of the targets for detection using a turbidimeter, and 2000 copies for detection with a visible color change [42]. In other reports, a LAMP system targeting the *rim*-encoding 16S rRNA-processing protein for detection of *M*. *tuberculosis* and *M*. *bovis* detected as low as 200 copies [43]. Our results therefore indicate that the newly established LAMP assay has a superior limit of detection compared to previously described systems. In addition, the LAMP can be used together with other conventional MTC identification methods, such as MGIT culture, to enhance detection of *M*. *bovis* infections. Furthermore, as well as its application in human disease, our LAMP could be employed to detect *M*. *bovis* from cattle samples after postmortem assisting in tracing back sources of infection and the implementation of appropriate control measures.

Our established LAMP can easily be integrated for use in resource limited settings due to its properties. It employs simple procedures that involve mixing the reaction reagents in a single tube before incubating at a constant temperature (66°C) for 40 min. Furthermore, the incubation of LAMP reagents doesn't require the use of expensive equipment or a standardized laboratory; a heat block or water bath is sufficient, and results can be visualized with the naked eye, eliminating the need for procedures such as gel electrophoresis and photographic imaging as is the case with PCR-based methods.

One major challenge to enzymatic-based methods is cold chain maintenance and storage of reagents in resource limited areas. To overcome this, Hayashida and others [24,44] used dried LAMP reagents, facilitating reagents' storage for an extended period without the need to maintain a cold chain. We plan to explore this option for our next study and further optimize the dry LAMP method for direct use against sputum samples and cattle post-mortem samples in a field setting. A further consideration in zoonotic TB is the emerging appreciation of the role of *Mycobacterium orygis* in human and bovine disease, particularly in South Asia [32,45,46]. Hence, further development of our LAMP system could allow for differential identification of *M*. *orygis* vs *M*. *bovis*, for example, by targeting the distinct RD12 deletion locus in *M*. *orygis* [47].

In conclusion, we have established a LAMP system for specific detection of *M*. *bovis* by targeting the RD4 deletion. The established LAMP showed high specificity and sensitivity, with ease and use coupled with rapid detection time. This new RD4 LAMP assay employs simple procedures and does not require expensive equipment, such as thermocyclers. Additionally, one reaction cycle of the RD4 LAMP would cost ~US$1–2, hence it is a cheaper approach as compared to other NAATs. Our future development work will focus on establishing a dry LAMP kit to facilitate transport and storage, and evaluate it on field samples.

## Supporting information

S1 Table Clinical and field isolates of *M*. *bovis* and *M*. *tuberculosis* used in this study. (XLSX)

S1 Fig Enzymatic digestion of LAMP products. (TIFF)
