# Supplementary figures and images for: Development of a loop-mediated isothermal amplification (LAMP) method for specific detection of Mycobacterium bovis
Source: PLoS Negl Trop Dis. 2021 Jan 25;15(1):e0008996. doi: 10.1371/journal.pntd.0008996 (PMC7833227; doi:10.1371/journal.pntd.0008996)

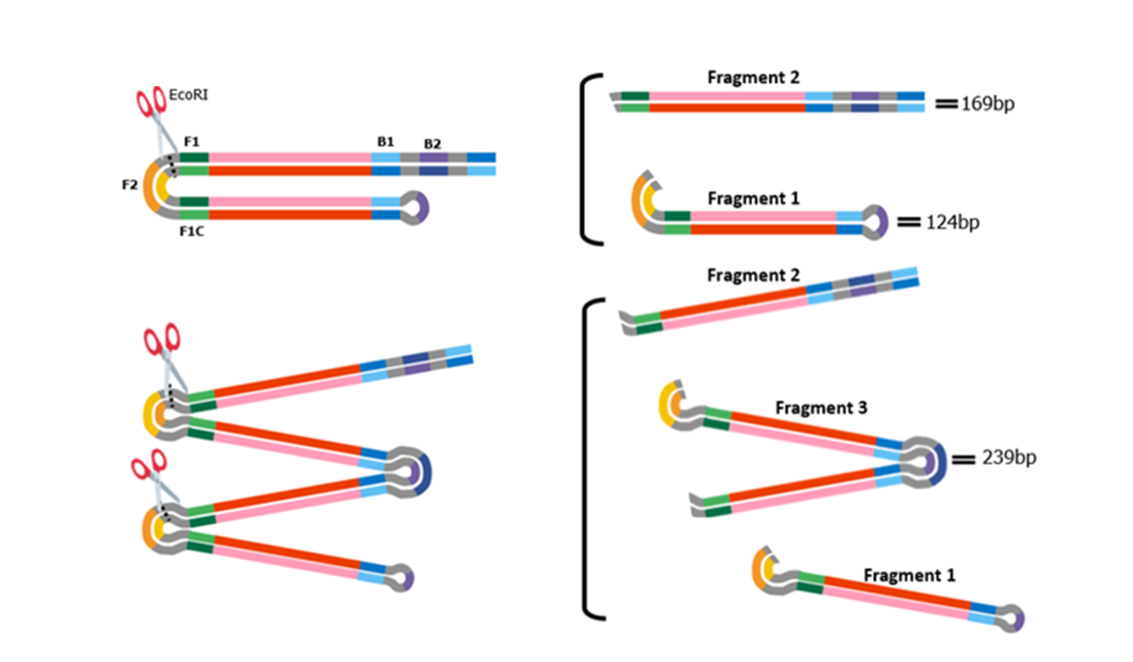

Supplement: S1 Fig — (TIFF) [file pntd.0008996.s002.tiff]
